# Sanicula Spring Water

**Published:** 1890-08

**Authors:** J. G. Gundlach

**Affiliations:** Prover of Sanicula Mineral Spring Water; Spokane Falls, Washington


### SANICULA SPRING WATER.

EDITORS HOMŒOPATHIC PHYSICIAN:—Have just read Dr. D. W. Clausen's timely caution regarding the Sanicula Mineral Spring Water in the June HOMŒOPATHIC PHYSICIAN. Please permit me to say further in this matter that some three months ago I received letters from the well-known pharmacy house of Bœricke & Tafel, of Philadelphia, asking for information about the Sanicula, how to secure the remedy, etc. In my reply I told them what the remedy was and how I had proved and obtained it, making them a proposition to this effect: If they would agree to compile and publish all the non-published matter of Sanicula and publish it in *The Homœopathic Recorder*, which they claim is devoted to introduction of new remedies, I would give them grafts of all the original potencies I have made by my own hand at the time of proving, I having retained them from the 9th to the 100th, so giving them complete control of this "new and highly valuable remedy," an offer which I thought they would gladly accept, but this they declined with thanks, saying, "we cannot use grafts." Now whether the above house is the same that Dr. R. B. Johnstone got Sanicula of, I, of course, do not know. Yet I hope not. Now to prevent any further trouble of this kind, I will say that Sanicula is only a name given to a mineral-water spring by myself. The springs are situated in Ottawa, Ill., and were formerly known as the Ottawa Mineral Springs.

The chemical analysis of the spring may be found in the January *Medical Advance*, of 1885, also in the *I. H. A. Trans.*, page 129, 1887, along with the last report of proving and clinical verifications of great importance. The proving was made on myself and family, using the water daily for some fifteen months (never, I may say, will such a proving be made again). The potencies I have were made during the winter months, while the river was at its lowest, as the spring is situated on the bank of the river, giving the water in its *strongest* form. (For this, gentlemen, "*Is water, as is water.*") "I thank you, Jew, for that word." (See *Med. Era*, February, 1885. "A needed reform.") I made my potency as follows: Filling a new vial direct from the spring, returning to the office, I poured out most of its contents; to that which remained I added about one hundred drops of distilled water, and after succussing, repeated the above process up to the 100th potency, retaining the intermediate potencies of 9th, 18th, and 30th. Dr. Berridge, of England, wrote me and I sent him grafts of the 9th. This he had Dr. Skinner potentize to the CM on his machine, sending me in return grafts of the 200, 500, 1M, 5M, 10M, 20M, 50M, and CM potencies. Of these I have used mostly the 10M, having placed it among the polychrests of my case, and in my practice find indications for it as common as those of Bell., Bry., or Puls. It saves me a great deal of talking or zigzaging, as our Dr. Lippe used to say.

I have supplied many with grafts, and it gives me great satisfaction to read of its verification. Will at any time furnish grafts to any desiring to use the remedy. I would repeat what I wrote to Messrs. Bœricke & Tafel,—especially as they have said that they could not use grafts—that the proving was made in 1884, and these potencies at the time of proving. Six years have since then passed, and the springs, for all that we know, may have changed their constituency in this time. I should at least not trust a potency made from the present conditions without a new proving.

J. G. Gundlach, M. D.,
(Prover of Sanicula Mineral Spring Water)
Spokane Falls, Washington.

